# Representativeness of a digitally engaged population and a patient organisation population with rheumatoid arthritis and their willingness to participate in research: a cross-sectional study

**DOI:** 10.1136/rmdopen-2018-000664

**Published:** 2018-06-20

**Authors:** Ruth Costello, Clare Jacklin, Matthew Jameson Evans, John McBeth, William G Dixon

**Affiliations:** 1 Arthritis Research UK Centre for Epidemiology, Division of Musculoskeletal and Dermatological Sciences, School of Biological Sciences, The University of Manchester, Manchester, UK; 2 National Rheumatoid Arthritis Society, Berkshire, UK; 3 HealthUnlocked (Everything Unlocked), London, UK; 4 NIHR Manchester Musculoskeletal Biomedical Research Unit, Central Manchester University Hospitals NHS Foundation Trust, Manchester Academic Health Science Centre, Manchester, UK; 5 Health eResearch Centre, Manchester Academic Health Science Centre, The University of Manchester, Manchester, UK

**Keywords:** rheumatoid arthritis, epidemiology, patient perspective.

## Abstract

**Objectives:**

To describe (1) the representativeness of (a) users of an online health community (HealthUnlocked.com (HU)) with rheumatoid arthritis (RA) and (b) paid members of an RA patient organisation, the National Rheumatoid Arthritis Society (NRAS), compared with the general RA population; and (2) the willingness of HU users with RA to participate in types of research (surveys, use of an app or activity tracker, and trials).

**Methods:**

A pop-up survey was embedded on HU to determine the characteristics of users and their willingness to participate in research. An anonymous data set of NRAS member characteristics was provided by the NRAS (N=2044). To represent the general RA population, characteristics of people with RA were identified from the Clinical Practice Research Datalink (CPRD) (N=20 594). Cross-sectional comparisons were made across the three groups.

**Results:**

Compared with CPRD, HU respondents (n=615) were significantly younger (49% aged below 55 years compared with 23% of CPRD patients), significantly more deprived (21% in the most deprived Townsend quintile compared with 12% of CPRD patients) and had more recent disease, with 62% diagnosed between 2010 and 2016 compared with 37% of CPRD patients. NRAS members were more similar to the CPRD, but significantly under-represented those aged 75 years or over and over-represented those aged 55–75 years compared with the CPRD. High proportions of HU users were willing to participate in future research of all types.

**Conclusions:**

NRAS members were broadly representative of the general RA population. HU users were younger, more deprived and more recently diagnosed. HU users were willing to participate in most types of research.

**Key messages**

**What is already known about this subject?**

- Studies are starting to recruit participants online and through patient organisations, but we do not know how representative these groups are.

**What does this study add?**

- Patient organisation members with rheumatoid arthritis (RA) were broadly representative of the general RA population, and online health community (OHC) users with RA were younger, more recently diagnosed and from more deprived areas.
- A high proportion of OHC users were willing to take part in all types of research (surveys, use of an app or activity tracker, and trials).

**How might this impact on clinical practice?**

- Future studies may be able to recruit more efficiently from OHCs and patient organisations with confidence in how these populations represent the study population.

## Introduction

Large population studies often require significant numbers of participants to generate enough statistical power. This often requires multisite recruitment through rheumatology departments. A study of trials conducted in 2002–2008 found only 55% recruited to their prespecified sample size.^1^ This leads to an underpowered study and possible inconclusive results.

Study recruitment may be improved in both numbers and efficiency by recruiting patients directly. This may be coordinated via patient organisations or, as patients are increasingly online,^2^ through the internet. For example, studies have recruited through social media,^3 4^ recruited through online forums,^5–7^ advertised on health websites^4^ or advertised based on health-related search terms on Google.^8^ However the representativeness of online health communities (OHCs) and patient organisations, particularly in a rheumatoid arthritis (RA) population, is not clear.

The aims of this study were to describe (1) the representativeness of paid members of a patient organisation with prevalent RA and users of an OHC with RA when compared with the general RA population, and (2) the types of studies that OHC users with RA would participate in.

## Methods

### Design

This cross-sectional study compared the characteristics of adults with RA from the National Rheumatoid Arthritis Society (NRAS) members who had paid for membership and visitors to the NRAS community group on HealthUnlocked.com (HU) with adults with RA identified from the Clinical Practice Research Datalink (CPRD), a database of anonymised UK primary care electronic medical records. As the CPRD is broadly representative of the UK population,^9^ adults with RA identified from the CPRD were considered representative of adults with RA in the UK.

### Patient organisation population

The NRAS is a patient organisation for people living with RA. When people join NRAS or renew their membership, they can provide demographic and medical information. An anonymised data set of all members, past and present up until 1 May 2016, was provided by the NRAS. For consistency with the other data sets, and to avoid selection bias, only current NRAS members were used. The data set contained (self-reported) year of RA diagnosis, ethnicity, current age, gender, employment status, and ever use of disease-modifying antirheumatic drugs (DMARDs), biologics and glucocorticoids (GC). To be included in the analyses, respondents had to be residents in the UK to allow comparison with the other UK data sets.

### HU population

HU is Europe’s largest OHC, with over 4.5 million visitors per month.^10^ The NRAS has a community group on HU for people with RA with, on average, 169 000 visitors per month. Anybody can visit the NRAS community on HU irrespective of a diagnosis of RA, NRAS membership or following the NRAS HU community. As people join HU without providing demographic information, a survey was developed to determine self-reported RA diagnosis, year of RA diagnosis, medications used, willingness to participate in different types of research (including questionnaires of varying durations, using an app, wearing an activity tracker and different types of trial), demographics (age, gender, employment, postcode and ethnicity) and the types of electronic devices owned (details of survey development in online supplementary file 1). After review by a combined patient and public involvement group and agreement with the NRAS, the finalised survey (online supplementary figure 1) was embedded in all posts within the NRAS HU community and popped up for completion when these posts were viewed by someone with a UK IP address. Prior to starting the survey, respondents confirmed they were over 18 years of age. The survey then started with an eligibility question to determine self-reported RA. The survey started on 6 May 2016 and was live for 3 months or until 1000 people had completed the survey, whichever was soonest. Postcode was converted to Townsend Deprivation Index^11^ by a health data scientist outside of the research team prior to analysis.

10.1136/rmdopen-2018-000664.supp2 Supplementary data

10.1136/rmdopen-2018-000664.supp1 Supplementary data

### CPRD population

A prevalent cohort of patients with a diagnosis of RA prior to 1 June 2016 was identified using a validated algorithm.^12^ Eligibility criteria were (1) aged 18 years or over at RA diagnosis, (2) registered at a practice on 1 May 2016 and (3) data met the CPRD quality standards.^9^ Age, gender, year of RA diagnosis, ethnicity, ever DMARD and GC use, and Townsend Deprivation Index (for practices that consented to linkage) were identified for these patients (covariate definitions in online supplementary file 1).

### Analysis

For each data set, the characteristics were categorised and tabulated to match the HU survey responses to allow comparison between data sets. A Z-test for the difference in proportions within each category of each characteristic was calculated comparing NRAS with CPRD, and HU with CPRD, where CPRD data were available. The characteristics of those who would definitely or probably take part in each type of research are reported. Logistic regression was used to identify any characteristics that were independently associated with definite or probable participation in each type of research.

### Missing data

To be included in this analysis, individuals had to have information on at least age and gender. For CPRD employment status was available for less than 5% of patients so it was not used in this analysis. For NRAS members, postcode and therefore Townsend Deprivation Index were unavailable. For all variables, except age and gender, when the variable was available for the data set, the percentage of missing data is reported.

## Results

### Data sets

#### NRAS

The NRAS provided a data set of 4505 current and past members. Of those, 1498 were not currently members, 22 were from overseas and 941 did not have information on age and gender, resulting in a data set of 2044 current members with RA.

#### HealthUnlocked survey

The HU survey was live for 74 days between 6 May 2016 and 12 August 2016 and had 100 112 pop-ups to unique IP addresses. There were 2647 pop-ups clicked, 900 respondents agreed to take part, 750 respondents were eligible, and 135 did not provide age and gender, resulting in 615 respondents available for analysis. Recruitment was steady with an average of 12 responses per day.

#### CPRD

Of 4 776 441 people in the CPRD, there were 20 594 (0.43%) patients with a diagnosis of RA on 1 June 2016.

### Characteristics

**Figure 1:**
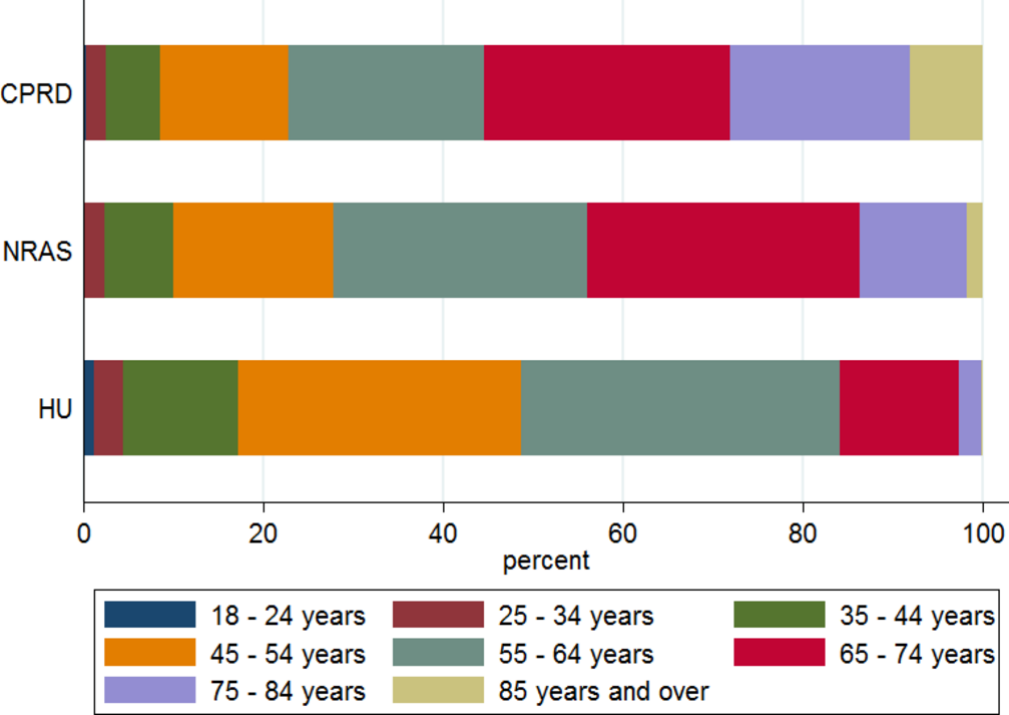
Proportions in each age group by data set. CPRD, Clinical PracticeResearch Datalink; HU, HealthUnlocked.com; NRAS, National RheumatoidArthritis Society.

Table 1 and figure 1 show that NRAS members had a reasonably similar age distribution to patients with RA from the CPRD up to age 55. After this age there were statistically significant differences in proportions, with an over-representation of people aged 55–75 years and an under-representation of people aged 75 years and over in NRAS members. HU users were a significantly younger population compared with the CPRD, with fewer responders aged 65 years or over. Both NRAS and HU were predominantly female, with significantly higher proportions (~85%) compared with CPRD (70%). HU users had shorter disease duration, with significantly more respondents diagnosed between 2010 and 2016 (62%) compared with CPRD participants (37%), while NRAS members has a longer disease duration, with significantly fewer people diagnosed between 2010 and 2016 (25%). HU responders had a significantly higher proportion of people from more deprived areas (most deprived Townsend quintile: HU: 22% vs CPRD 12%) and significantly less from affluent areas (least deprived Townsend quintile: HU: 18% vs CPRD 23%) (data not available for NRAS members). All DMARDs had significantly more ever use in both HU and NRAS compared with CPRD.

**Table 1:** Characteristics of patients with RA who are NRAS members, or who responded to a survey on HU and those identified from CPRD

	CPRD (N=20 594)	NRAS (N=2044)	Difference in proportion compared with CPRD (95% CI)	P values	HU(N=615)	Difference in proportion compared with CPRD (95% CI)	P values
	n (%)	n (%)			n (%)		
Age (years)							
18–34	499 (2.4)	37 (1.8)	0.6 (−0.002 to 1.2)	0.08	26 (4.2)	−1.8 (−3.4 to −0.02)	0.005
35–44	1249 (6.1)	129 (6.3)	−0.2 (−1.3 to 0.9)	0.66	79 (12.9)	−6.8 (−9.4 to −4.1)	<0.001
45–54	2936 (14.3)	311 (15.2)	−1 (−3 to 0.7)	0.24	195 (31.7)	−17.5 (−21.2 to −13.7)	<0.001
55–64	4484 (21.8)	568 (27.8)	−6 (−8 to −4)	<0.001	218 (35.5)	−13.7 (−17.5 to −9.9)	<0.001
65–74	5643 (27.4)	709 (34.7)	−7 (−9 to −5)	<0.001	81 (13.2)	14.2 (11.5 to 17.0)	<0.001
75 and over	5783 (28)	290 (14.2)	13.9 (12.3 to 15.5)	<0.001	16 (2.6)	25.4 (24.1 to 26.9)	<0.001
Gender							
Female	14 440 (70.1)	1728 (84.5)	−0.18 (−0.21 to −0.16)	<0.001	544 (88.5)	−18.3 (−20.9 to −15.7)	<0.001
Year of RA diagnosis							
<1990	34 (0.2)	274 (14.5)	−14.3 (−15.9 to −12.7)	<0.001	39 (6.3)	−6.2 (−8.1 to −4.2)	<0.001
1990–1994	869 (4.2)	122 (6.5)	−2.2 (−3.4 to −1.1)	<0.001	22 (3.6)	0.6 (−0.9 to 2.1)	0.43
1995–1999	1756 (8.5)	178 (9.4)	−0.9 (−2.3 to 0.5)	0.19	32 (5.2)	3.3 (1.5 to 5.1)	0.004
2000–2004	4336 (21.1)	304 (16.1)	5.0 (3.2 to 6.7)	<0.001	46 (7.5)	13.5 (11.4 to 15.7)	<0.001
2005–2009	5962 (29)	540 (28.6)	0.4 (−1.7 to 2.5)	0.72	95 (15.5)	13.5 (10.6 to 16.4)	<0.001
2010–2016	7637 (37.1)	473 (25)	12.1 (10 to 14.1)	<0.001	381 (62)	−24.9 (−28.8 to −21.0)	<0.001
Missing	0	153			0		
Employment status							
Full-time employed		543 (24.1)			387 (23.6)		
Part-time employed		418 (18.5)			288 (17.5)		
Unemployed		304 (13.5)			280 (17)		
Retired		698 (30.9)			577 (35.1)		
Retired due to arthritis		151 (6.7)			83 (5.1)		
Not working due to ill health		142 (6.3)			28 (1.7)		
Missing	NA	401			0		
Ethnicity							
White	8931 (93.9)	1572 (98.4)	−4.5 (−5.3 to −3.7)	<0.001	578 (94.6)	−0.73 (−2.6 to 1.1)	0.47
Mixed	44 (0.5)	5 (0.3)	0.1 (−0.2 to 0.5)	0.40	8 (1.3)	−0.8 (−1.8 to 0.06)	0.005
Asian	315 (3.3)	12 (0.8)	2.6 (2.0 to 3.1)	<0.001	11 (1.8)	1.5 (0.3 to 2.6)	0.04
Black	147 (1.6)	5 (0.3)	1.2 (0.9 to 1.6)	<0.001	13 (2.1)	−0.58 (−1.8 to 0.6)	0.26
Other	77 (0.8)	4 (0.3)	0.6 (0.3 to 0.9)	0.02	1 (0.2)	0.6 (0.3 to 1.0)	0.08
Missing	11 080	446			2		
Townsend Deprivation Index							
1 (least deprived)	2807 (23.1)				103 (18.3)	4.9 (1.6 to 8.1)	0.007
2	2949 (24.3)				112 (19.9)	4.4 (1 to 7.8)	0.016
3	2543 (20.9)				107 (19)	2.0 (−1.3 to 5.3)	0.26
4	2329 (19.2)				119 (21.1)	−1.9 (−5.4 to 1.5)	0.26
5 (most deprived)	1514 (12.5)				123 (21.8)	−9.3 (−12.8 to -5.9)	<0.001
Missing	8452	NA			49		
Ever taken methotrexate							
Yes	14 553 (70.7)	1783 (87.2)	−16.6 (−18.1 to −15.0)	<0.001	511 (84.9)	−14.2 (−17.1 to −11.3)	<0.001
Missing or unknown	0	0			13		
Ever taken sulfasalazine							
Yes	9742 (47.3)	1064 (52.1)	−4.7 (−7 to −2.5)	<0.001	339 (56.9)	−9.6 (−13.6 to −5.5)	<0.001
Missing or unknown	0	0			19		
Ever taken hydroxychloroquine							
Yes	8255 (40.1)	883 (43.2)	−3.1 (−5.4 to −0.9)	0.006	359 (60.1)	−20 (−24 to −16)	<0.001
Missing or unknown	0	0			18		
Ever taken leflunomide							
Yes	2576 (12.5)	378 (18.5)	−6 (−7.7 to −4.2)	<0.001	113 (19)	−6.5 (−9.7 to −3.3)	<0.001
Missing or unknown	0	0			21		
Ever taken DMARDs*							
Yes	18 683 (90.7)	1956 (95.7)	−5 (−6 to −4)	<0.001	537 (93.4)	−2.7 (−4.7 to −0.6)	0.029
Missing or unknown	0	0			106		
Ever taken glucocorticoids							
Yes	11 889 (57.7)	351 (17.2)	40.6 (38.8 to 42.3)	<0.001	368 (61.7)	−4 (−8 to −0.05)	0.05
Missing or unknown	0	0			19		
Ever taken biologics							
Yes		897 (43.9)			196 (32.9)		
Missing or unknown	20 594	0			20		

*Ever taken DMARDs is based on the ever taken methotrexate, ever taken sulfasalazine, ever taken hydroxychloroquine and ever taken leflunomide data.

CPRD, Clinical PracticeResearch Datalink; DMARD, disease-modifyingantirheumatic drug; HU, HealthUnlocked.com; NA, not available; NRAS, National RheumatoidArthritis Society; RA, rheumatoid arthritis.

### Participation in future research (HealthUnlocked only)

HU responders commonly reported they were definitely or probably willing to take part in future research, particularly questionnaires, with 89% reporting willingness to complete a questionnaire of 10 min. A lower proportion reported willingness to use an app (63%) compared with wearing an activity tracker (74%). Half of the respondents reported willingness to take part in a drug trial via the internet or with site visits. When stratified by age, overall those over 65 years of age reported less willingness to take part in all research types. When stratified by gender, men reported more willingness to use an app while women reported more willingness to wear an activity tracker (table 2). The most striking result from multivariate logistic regression showed that participants over 45 years of age were significantly less willing to use an app compared with those aged 18–34 years. Those aged 45–54 years had a 92% lower odds of using an app (OR: 0.08 (95% CI 0.01 to 0.58)), and those aged 75 years and over had 98% lower odds of using an app compared with those aged 18–34 years (OR: 0.02 (95% CI 0.002 to 0.23)). There were no statistically significant differences by gender (online supplementary table 1).

**Table 2:** Proportion of HealthUnlocked users who would definitely or probably take part in types of research

Characteristics	Type of research						
	Complete questionnaires				Participate in trials		
	Single 10 min	Multiple questionnaires over months?	Using an app	Wearing an activity tracker	Non-drug treatment	Drug treatment via internet alone	Drug treatment with site visits
Total	546 (89.1)	503 (82.1)	387 (63.1)	453 (73.9)	401 (65.4)	305 (49.8)	327 (53.3)
Age category (years)							
18–34	23 (88.5)	19 (73.1)	25 (96.2)	20 (76.9)	18 (69.2)	12 (46.2)	15 (57.7)
35–44	73 (92.4)	69 (87.3)	60 (76)	65 (82.3)	55 (69.6)	48 (60.8)	51 (64.6)
45–54	172 (88.2)	155 (79.5)	123 (63.1)	145 (74.4)	134 (68.7)	108 (55.4)	108 (55.4)
55–64	201 (92.2)	184 (84.4)	133 (61)	157 (72)	138 (63.3)	100 (45.9)	111 (50.9)
65–74	63 (77.8)	65 (80.3)	41 (50.6)	58 (71.6)	48 (59.3)	32 (39.5)	38 (46.9)
75 and over	16 (100)	13 (81.3)	6 (37.5)	9 (56.3)	9 (56.3)	5 (31.3)	5 (31.3)
Gender							
Female	483 (89.1)	446 (82.3)	337 (62.2)	408 (75.3)	353 (65.1)	267 (49.3)	288 (53.1)
Employment status							
Full-time employment	140 (89.7)	122 (78.2)	102 (65.4)	119 (76.3)	105 (67.3)	78 (50)	72 (46.2)
Part-time employment	115 (88.5)	102 (78.5)	75 (57.7)	90 (69.2)	78 (60)	64 (49.2)	71 (54.6)
Unemployed	21 (87.5)	20 (83.3)	16 (66.7)	18 (75)	16 (66.7)	12 (50)	16 (66.7)
Retired	101 (83.5)	98 (81)	67 (55.4)	84 (69.4)	69 (57)	47 (38.8)	53 (43.8)
Retired due to arthritis	59 (86.8)	59 (86.8)	44 (64.7)	50 (73.5)	43 (63.2)	34 (50)	40 (58.8)
Not working due to ill health	110 (96.5)	102 (89.5)	83 (72.8)	92 (80.7)	90 (79)	70 (61.4)	75 (65.8)
Missing	2	2	2	2	2	2	2
Ethnicity							
White	518 (89.6)	481 (83.2)	362 (62.6)	426 (73.7)	373 (64.5)	291 (50.4)	311 (53.8)
Mixed	8 (100)	7 (87.5)	6 (75)	7 (87.5)	6 (75)	5 (62.5)	5 (62.5)
Asian	7 (63.6)	3 (27.3)	6 (54.6)	7 (63.6)	7 (63.6)	3 (27.3)	3 (27.3)
Black	10 (76.9)	9 (69.2)	10 (76.9)	11 (84.6)	13 (100)	5 (38.5)	6 (46.2)
Other	1 (100)	1 (100)	1 (100)	1 (100)	0 (0)	1 (100)	1 (100)
Missing	4	4	4	4	4	4	4
Townsend Deprivation Index							
1	96 (93.2)	86 (83.5)	60 (58.3)	73 (70.9)	58 (56.3)	52 (50.5)	50 (48.5)
2	103 (92)	96 (85.7)	72 (64.3)	92 (82.1)	84 (75)	58 (51.8)	58 (51.8)
3	100 (93.5)	86 (80.4)	71 (66.4)	84 (78.5)	78 (72.9)	64 (59.8)	66 (61.7)
4	107 (89.9)	102 (85.7)	73 (61.3)	77 (64.7)	68 (57.1)	54 (45.4)	63 (52.9)
5	104 (84.6)	101 (82.1)	87 (70.7)	97 (78.9)	91 (74)	59 (48)	71 (57.7)
Missing	51	51	51	51	51	51	51

10.1136/rmdopen-2018-000664.supp3 Supplementary data

## Discussion

This study shows that people with RA who were NRAS members were reasonably representative of the general RA population, although fewer were aged 75 years or over. HU visitors were a younger RA population, with more recent disease and more deprivation than the general RA population. Most respondents from the OHC were willing to take part in studies with lower burden. More than half were willing to take part in any type of study including a drug trial via the internet. Younger participants were more willing to use an app. We have also demonstrated that over 600 responses to a short questionnaire can be collected over a short period of time using pop-ups within an OHC.

Recruiting online through HU was a straightforward and less labour-intensive method of recruiting a reasonably large sample of respondents in a short space of time. Once the survey had been designed and then implemented by HU, other than monitoring the numbers of surveys completed, it did not require further work by the study team as data were automatically captured. This contrasts to more traditional methods where a person is required to collect data for each survey throughout data collection. Ninety per cent of those aged 55–64 reported they recently used the internet in a UK national survey.^13^ This is seen in this study with good representation of people aged 45–65 years in our sample. Those aged over 75 years were not well represented and may be expected given that one in four of those aged 75 or over are online,^13^ and this may impact the generalisability of study results and would need to be considered by investigators designing studies. For example, if the disease of interest affects elderly people, studies using HU may wish to consider additional recruitment sources to ensure that elderly patients are represented. Conversely, if investigators are interested in deprivation or people recently diagnosed with RA, then HU may be a good source of participants.

Few studies have looked specifically at the representativeness of members of a patient organisation or internet users with RA. A study including a group of patients with RA found that internet users were younger, more educated and more commonly employed compared with those who did not use the internet for health.^14^ In this study HU responders were younger, with a similar proportion employed compared with NRAS members, although we did not have CPRD as comparison. Although there was no CPRD comparison, the proportion who had ever taken biologics was high in both HU and NRAS compared with the reported UK estimates of 11%–16%.^15 16^ This may indicate that both NRAS and HU respondents have more severe disease requiring biologics, and this may be why they are using HU or NRAS. However as we do not have disease activity measures, we cannot be sure of this.

There were some limitations to this study. RA diagnosis in the CPRD relies on Read (diagnosis) codes and drug codes so there may be some misclassification. The prevalence rate of RA (0.43%) is lower than the 1% prevalence otherwise estimated,^17^ which supports some misclassification. RA diagnosis relied on self-report for both the NRAS and HU, so there may have been some misclassification; however, as these groups are both specific for RA, it is likely that any misclassification would be small. NRAS characteristics relied on members providing personal details: there may be some selection bias if those who gave information were different from those who did not. Further to this, as NRAS membership requires payment, there may be some selection bias in that NRAS members may be less deprived than the general population; however, we were unable to capture the Townsend Deprivation Index for this group. There may be some HU respondents who were NRAS members also; 114 HU respondents indicated they were NRAS members. However, it was not possible to cross-reference the NRAS and HU data sets. The HU characteristics reflect the characteristics of those who completed the survey, so may not be representative of all HU users, but does provide insight into the characteristics of those willing to join studies via this route. Although we did not survey NRAS paid members about their willingness to participate in research, recent experience demonstrates that patients with RA, both members and non-members, are responsive to participating in research following outreach from the NRAS.

This study gives an indication of the representativeness of groups that investigators may consider using to recruit people with RA to studies, while also demonstrating the feasibility of recruitment from OHCs. People in OHCs are willing to take part in many types of research, with the proportion declining as the burden of the research increases.
