# Supplementary material for: Representativeness of a digitally engaged population and a patient organisation population with rheumatoid arthritis and their willingness to participate in research: a cross-sectional study
Source: RMD Open. 2018 Jun 20;4(1):e000664. doi: 10.1136/rmdopen-2018-000664 (PMC6018858; doi:10.1136/rmdopen-2018-000664)
Supplement: Supplementary data [file rmdopen-2018-000664supp002.pdf]

Supplementary 1

Survey development

A short survey was drafted by the research team to collect information on: RA diagnosis, NRAS membership and following the NRAS community on HU , the year of RA diagnosis, medications currently used and used in the past, willingness to participate is different types of research, demographics (age, gender, employment, postcode and ethnicity) and the types of electronic devices owned. The survey was reviewed by epidemiologists, rheumatologists and a representative from NRAS that resulted in updates to the language of the survey to ensure it was easily understandable, additional drug brand examples and additional options for some questions. The survey was then tested for how understandable it was by an existing Manchester-based musculoskeletal research user group, which comprises of 12 people either with musculoskeletal disease or carers of someone with musculoskeletal disease. This testing did not result in any changes to the survey.

CPRD covariate definitions

Ethnicity was identified, where present, using Read codes. If a patient had more than one ethnicity listed on their records ethnicity was set to missing.

Employment status was identified using Read codes recorded within the five years prior to 1st May 2016. If there were multiple records of employment status the closest to 1st May 2016 was used.

DMARD and glucocorticoid use was identified using product codes. If a person had a prescription for a drug since diagnosis they were defined as an ever user of the medication.

Socioeconomic status (SES) was available for those practices that consented to linkage. For those practices a Townsend score was provided for the patients within those practices.
