# Supplementary material for: Representativeness of a digitally engaged population and a patient organisation population with rheumatoid arthritis and their willingness to participate in research: a cross-sectional study
Source: RMD Open. 2018 Jun 20;4(1):e000664. doi: 10.1136/rmdopen-2018-000664 (PMC6018858; doi:10.1136/rmdopen-2018-000664)
Supplement: Supplementary data [file rmdopen-2018-000664supp001.pdf]

Survey of people with Rheumatoid arthritis who visit HealthUnlocked:
Who are you and how willing are you to take part in future research?

We are interested in using HealthUnlocked to identify people with rheumatoid arthritis who could participate in research studies. Before we do this we'd like to know two things. First, how do HealthUnlocked members and visitors with rheumatoid arthritis compare to the general rheumatoid arthritis population. And, second, what types of research would visitors to HealthUnlocked be prepared to take part in. We'd like to invite you to take this short survey about yourself, your rheumatoid arthritis and the types of future research you may be willing to participate in.

Before you decide whether you want to complete the survey, it is important for you to understand why the survey is being done and what your participation will involve. Please take time to read the following information carefully:

- The survey is being funded by the Arthritis Research UK and conducted by the University of Manchester, in partnership with HealthUnlocked.
- All information you provide will be anonymous and treated in the strictest confidence and according to legal and ethical guidelines of the UK Data Protection Act 1998.
- The information will be stored at the University of Manchester for 10 years.
- If you decide to take part you are still free to withdraw at any time during the survey. However, as data collection is anonymous and sent when the survey is completed, your information cannot be identified to withdraw after survey completion.

The survey should take no more than 5 minutes to complete and is entirely voluntary.

☐ **I confirm I have read and understood the statements above and agree to take part in the study.**

☐ **I confirm I am aged 18 years or over.**

Have you ever been told by a doctor that you have or had Rheumatoid Arthritis (RA)?

◯ Yes

◯ No

Are you a member of the National Rheumatoid Arthritis Society (NRAS)?

i.e. you pay an annual or paid membership subscription to the society.

◯ Yes

◯ No

Do you follow the NRAS community on HealthUnlocked?

Followers receive community updates and can contribute

- ◯ Yes
- ◯ No

Roughly, what year was your Rheumatoid Arthritis (RA) diagnosed?

- ◯ Before 1990
- ◯ 1990-1994
- ◯ 1995-1999
- ◯ 2000-2004
- ◯ 2005-2009
- ◯ 2010-2015

Do you use, or have you ever used the following medications:

	Currently taking	Have taken in the past	Never taken	I don't know
**Methotrexate**<br>Also called Maxtrex or Metoject	◯	◯	◯	◯
**Sulfasalazine**<br>Also called Salazopyrin	◯	◯	◯	◯
**Leflunomide**<br>Also called Arava	◯	◯	◯	◯
**Hydroxychloroquine**<br>Also called Plaquinel	◯	◯	◯	◯
**Biological treatments**<br>Also called etanercept (Enbrel), infliximab (Remicade, Remsima or Inflectra), adalimumab (Humira), certolizumab (Cimzia), golimumab (Simponi), rituximab (Mabthera), abatacept (Orencia) or tocilizumab (Actemra/ Roactemra)	◯	◯	◯	◯
**Biosimilar biologic treatment**<br>Also called Remisima or Infectra.	◯	◯	◯	◯
**Glucocorticoids/ Corticosteroids/ steroids (tablets)**<br>Also called prednisolone or dexamethasone	◯	◯	◯	◯

To what extent would you be willing to join the following types of research study in the future?

	Definitely yes	Probably yes	Probably no	Definitely no
Single questionnaire up to 1 minute	○	○	○	○
Single questionnaire up to 5 minutes	○	○	○	○
Single questionnaire up to 10 minutes	○	○	○	○
Multiple questionnaires over a number of weeks	○	○	○	○
Multiple questionnaires over a number of months	○	○	○	○
Provide regular information via an app (mobile phone application)	○	○	○	○
Wear an activity tracker that would be sent to you	○	○	○	○
Testing of a non-drug treatment (e.g. online exercise programme, sleep intervention) requiring regular entries via internet (computer or mobile)	○	○	○	○
Testing of a new medicine requiring regular entries via internet (computer or mobile)	○	○	○	○
Trial of a medicine requiring repeated visits to a healthcare provider which may include blood tests, image tests e.g. xray, MRI, Ultrasound, biopsies etc.	○	○	○	○

What is your age group?

- ◯ 18-24
- ◯ 25-34
- ◯ 35-44
- ◯ 45-54
- ◯ 55-64
- ◯ 65-74
- ◯ 75-84
- ◯ over 85 years

What gender best describes you?

- ◯ Male
- ◯ Female

Please provide first three digits of your postcode:

What is your current employment status?

- ◯ Full-time employment
- ◯ Part-time employment
- ◯ Unemployed
- ◯ Retired
- ◯ Retired due to arthritis
- ◯ Not working due to ill health

Please indicate which ethnic group you consider yourself to belong to:

- ◯ White
- ◯ Mixed ethnic groups
- ◯ Asian or Asian British
- ◯ Black or Black British
- ◯ Other ethnic group

Do you own any of the following? Select all that apply (if any).

- ☐ Smartphone (e.g. iPhone, Samsung Galaxy)
- ☐ Activity tracker (e.g. Fitbit)
- ☐ Tablet computer (e.g.iPad, Google nexus)
- ☐ Laptop or desktop computer

Pressing next will submit your answers.

Thank you very much for agreeing to participate in this survey.
