# Supplementary material for: Representativeness of a digitally engaged population and a patient organisation population with rheumatoid arthritis and their willingness to participate in research: a cross-sectional study
Source: RMD Open. 2018 Jun 20;4(1):e000664. doi: 10.1136/rmdopen-2018-000664 (PMC6018858; doi:10.1136/rmdopen-2018-000664)
Supplement: Supplementary data [file rmdopen-2018-000664supp003.docx]

Supplemental table 1: Multivariable odds ratios (95% confidence intervals) for possibly or probably participating in each type of research (N=615).

|  | Type of research | | | | | | | | |
| --- | --- | --- | --- | --- | --- | --- | --- | --- | --- |
| Characteristic | Complete questionnaires | | Use an app | Wear an activity tracker | Participate in trials | | | | |
|  | Single 10 mins | Multiple questionnaires over months |  |  | Non-drug treatment | | Drug treatment via internet alone | | Drug treatment with site visits |
|  | Odds ratio (95% confidence interval) | | | | | | | | |
| Age |  |  |  |  |  |  | |  | |
| 18-34 years | Reference | Reference | Reference | Reference | Reference | Reference | | Reference | |
| 35-44 years | 2.66 (0.51, 13.87) | 3.07 (0.89, 10.65) | 0.13 (0.02, 1.06) | 1.36 (0.41, 4.56) | 0.93 (0.31, 2.81) | 1.82 (0.68, 4.88) | | 1.41 (0.52, 3.79) | |
| 45 - 54 years | 1.24 (0.31, 4.93) | 1.45 (0.5, 4.16) | 0.08 (0.01, 0.58) | 0.94 (0.31, 2.83) | 0.9 (0.32, 2.55) | 1.49 (0.6, 3.73) | | 0.95 (0.38, 2.38) | |
| 55 - 64 years | 2.42 (0.55, 10.58) | 1.86 (0.62, 5.52) | 0.06 (0.01, 0.48) | 0.84 (0.28, 2.56) | 0.78 (0.27, 2.23) | 0.94 (0.37, 2.37) | | 0.77 (0.3, 1.95) | |
| 65 - 74 years | 1.03 (0.19, 5.66) | 1.57 (0.4, 6.18) | 0.04 (0.005, 0.34) | 0.83 (0.22, 3.13) | 0.91 (0.27, 3.12) | 0.78 (0.25, 2.39) | | 0.83 (0.27, 2.56) | |
| 75 years and over | 1 (0, 0) | 1.37 (0.23, 8.01) | 0.02 (0.002, 0.23) | 0.43 (0.09, 2.12) | 0.8 (0.17, 3.78) | 0.60 (0.14, 2.62) | | 0.45 (0.1, 1.99) | |
| gender |  |  |  |  |  |  | |  | |
| Female | 0.61 (0.2, 1.82) | 1.1 (0.53, 2.29) | 0.59 (0.32, 1.08) | 1.52 (0.84, 2.73) | 0.64 (0.34, 1.18) | 0.65 (0.37, 1.14) | | 0.73 (0.41, 1.28) | |
| Year RA diagnosis |  |  |  |  |  |  | |  | |
| <1990 | Reference | Reference | Reference | Reference | Reference | Reference | | Reference | |
| 1990 – 1994 | 1.95 (0.19, 20.35) | 2.02 (0.46, 8.9) | 1.24 (0.36, 4.23) | 4.63 (0.88, 24.49) | 1.58 (0.47, 5.38) | 1.94 (0.59, 6.38) | | 1.35 (0.38, 4.78) | |
| 1995 – 1999 | 1.31 (0.21, 8.36) | 1.46 (0.44, 4.92) | 1.09 (0.38, 3.07) | 0.9 (0.31, 2.6) | 0.86 (0.3, 2.41) | 0.59 (0.21, 1.62) | | 0.47 (0.17, 1.32) | |
| 2000 – 2004 | 0.92 (0.19, 4.4) | 1.61 (0.52, 4.92) | 1.12 (0.43, 2.95) | 1.66 (0.58, 4.77) | 0.98 (0.37, 2.57) | 0.56 (0.22, 1.42) | | 0.29 (0.11, 0.76) | |
| 2005 – 2009 | 0.91 (0.24, 3.35) | 2.25 (0.84, 6.03) | 0.9 (0.4, 2.06) | 1 (0.42, 2.36) | 0.87 (0.38, 2) | 0.6 (0.27, 1.35) | | 0.49 (0.21, 1.13) | |
| 2010 – 2016 | 0.91 (0.27, 3.04) | 2.43 (1.03, 5.74) | 0.97 (0.46, 2.03) | 1.65 (0.75, 3.62) | 1.69 (0.79, 3.6) | 0.95 (0.46, 1.95) | | 0.67 (0.31, 1.42) | |
| Employment status |  |  |  |  |  |  | |  | |
| Full-time employed | Reference | Reference | Reference | Reference | Reference | Reference | | Reference | |
| Part-time employed | 0.69 (0.28, 1.68) | 1.05 (0.54, 2.02) | 0.72 (0.42, 1.24) | 0.8 (0.44, 1.44) | 0.81 (0.47, 1.4) | 0.92 (0.55, 1.54) | | 1.39 (0.83, 2.34) | |
| Unemployed | 0.97 (0.18, 5.18) | 2.82 (0.53, 14.96) | 1.34 (0.47, 3.79) | 1.48 (0.45, 4.87) | 1.97 (0.66, 5.92) | 1.29 (0.49, 3.38) | | 2.95 (1.04, 8.34) | |
| Retired | 0.56 (0.17, 1.89) | 1.38 (0.54, 3.53) | 1.3 (0.63, 2.68) | 1.18 (0.53, 2.63) | 0.74 (0.35, 1.56) | 1.02 (0.5, 2.06) | | 1.15 (0.57, 2.34) | |
| Retired due to arthritis | 0.42 (0.14, 1.24) | 1.72 (0.69, 4.28) | 1.25 (0.63, 2.5) | 1.13 (0.52, 2.43) | 0.95 (0.46, 1.94) | 1.33 (0.68, 2.6) | | 2.13 (1.08, 4.2) | |
| Not working due to ill health | 2.58 (0.78, 8.5) | 2.33 (1.08, 5) | 1.35 (0.76, 2.37) | 1.62 (0.85, 3.08) | 1.92 (1.04, 3.53) | 1.73 (1.02, 2.94) | | 2.27 (1.33, 3.89) | |
| Ethnicity |  |  |  |  |  |  | |  | |
| White | Reference | Reference | Reference | Reference | Reference | Reference | |  | |
| Mixed | 1 (0, 0) | 1.32 (0.14, 12.51) | 0.95 (0.17, 5.42) | 1.9 (0.21, 16.79) | 1.35 (0.25, 7.36) | 1.43 (0.32, 6.47) | | 0.93 (0.21, 4.22) | |
| Asian | 0.19 (0.04, 0.98) | 0.07 (0.02, 0.32) | 0.75 (0.17, 3.28) | 0.91 (0.18, 4.65) | 0.67 (0.16, 2.87) | 0.4 (0.1, 1.69) | | 0.31 (0.07, 1.32) | |
| Black | 0.35 (0.08, 1.5) | 0.49 (0.12, 2.02) | 1.41 (0.36, 5.61) | 1.43 (0.29, 7.12) | 1 (0, 0) | 0.55 (0.15, 2.03) | | 0.63 (0.18, 2.21) | |
| Other | 1 (0, 0) | 1 (0, 0) | 1 (0, 0) | 1 (0, 0) | 1 (0, 0) | 1 (0, 0) | | 1 (0, 0) | |
| Townsend deprivation index |  |  |  |  |  |  | |  | |
| 1 |  |  |  |  |  |  | |  | |
| 2 | 0.88 (0.31, 2.54) | 1.4 (0.65, 3.01) | 1.21 (0.68, 2.14) | 1.98 (1.02, 3.84) | 2.62 (1.43, 4.8) | 1.08 (0.62, 1.88) | | 1.19 (0.68, 2.07) | |
| 3 | 0.93 (0.3, 2.83) | 0.87 (0.42, 1.81) | 1.17 (0.66, 2.1) | 1.44 (0.76, 2.76) | 1.94 (1.07, 3.53) | 1.26 (0.72, 2.23) | | 1.59 (0.9, 2.82) | |
| 4 | 0.61 (0.22, 1.67) | 1.25 (0.58, 2.7) | 0.9 (0.51, 1.58) | 0.72 (0.39, 1.3) | 0.9 (0.52, 1.58) | 0.67 (0.38, 1.18) | | 1 (0.57, 1.75) | |
| 5 | 0.34 (0.13, 0.91) | 1.05 (0.5, 2.21) | 1.41 (0.78, 2.52) | 1.56 (0.82, 2.96) | 1.99 (1.1, 3.61) | 0.86 (0.49, 1.51) | | 1.44 (0.82, 2.54) | |
